# Designing eHealth that Matters via a Multidisciplinary Requirements Development Approach

**DOI:** 10.2196/resprot.2547

**Published:** 2013-06-24

**Authors:** Lex Van Velsen, Jobke Wentzel, Julia EWC Van Gemert-Pijnen

**Affiliations:** ^1^Center for eHealth Research and Disease ManagementDepartment of Psychology, Health, and TechnologyUniversity of TwenteEnschedeNetherlands; ^2^National Coordination Centre for Outbreak ManagementNational Institute for Public Health and the EnvironmentBilthovenNetherlands

**Keywords:** health care information systems, health informatics, requirements analysis, software design techniques, user-centered design

## Abstract

**Background:**

Requirements development is a crucial part of eHealth design. It entails all the activities devoted to requirements identification, the communication of requirements to other developers, and their evaluation. Currently, a requirements development approach geared towards the specifics of the eHealth domain is lacking. This is likely to result in a mismatch between the developed technology and end user characteristics, physical surroundings, and the organizational context of use. It also makes it hard to judge the quality of eHealth design, since it makes it difficult to gear evaluations of eHealth to the main goals it is supposed to serve.

**Objective:**

In order to facilitate the creation of eHealth that matters, we present a practical, multidisciplinary requirements development approach which is embedded in a holistic design approach for eHealth (the Center for eHealth Research roadmap) that incorporates both human-centered design and business modeling.

**Methods:**

Our requirements development approach consists of five phases. In the first, preparatory, phase the project team is composed and the overall goal(s) of the eHealth intervention are decided upon. Second, primary end users and other stakeholders are identified by means of audience segmentation techniques and our stakeholder identification method. Third, the designated context of use is mapped and end users are profiled by means of requirements elicitation methods (eg, interviews, focus groups, or observations). Fourth, stakeholder values and eHealth intervention requirements are distilled from data transcripts, which leads to phase five, in which requirements are communicated to other developers using a requirements notation template we developed specifically for the context of eHealth technologies.

**Results:**

The end result of our requirements development approach for eHealth interventions is a design document which includes functional and non-functional requirements, a list of stakeholder values, and end user profiles in the form of personas (fictitious end users, representative of a primary end user group).

**Conclusions:**

The requirements development approach presented in this article enables eHealth developers to apply a systematic and multi-disciplinary approach towards the creation of requirements. The cooperation between health, engineering, and social sciences creates a situation in which a mismatch between design, end users, and the organizational context can be avoided. Furthermore, we suggest to evaluate eHealth on a feature-specific level in order to learn exactly why such a technology does or does not live up to its expectations.

## Introduction

Requirements are the foundation of technology design. They describe what a technology should do, what data it should store or retrieve, what content it should display, and what kind of user experience it should provide. The development of requirements includes all the activities devoted to their identification, the communication of requirements to other developers, and their evaluation [1]. Involving end users and stakeholders in the creation of requirements has been shown to be a fruitful approach. It improves usability [2], prevents the inclusion of superfluous features [3], and can prevent the spending of money on bad design [2].

Within the literature on electronic health (eHealth) design, reports on the development of requirements are scarce. Coble et al [4] have reported on their experiences during the development of an information system for clinicians that displays their patients’ test results. Caligtan et al [5] discussed their creation of requirements for bedside information technology for patients. Thew et al [6] finally, have documented their experiences while creating requirements for geographic visualization tools for the epidemiology domain. Often, the creation of requirements is left to engineers who apply a technology-driven approach. However, The potential of eHealth technology can only be fully exploited when it is developed by a multi-disciplinary team who apply a human-centered approach that takes the specifics of the context (both organizational and that of the individual user) in which the technology is to be used into account [7,8]. This mismatch between context and technology has been recognized by the World Health Organization as the main reason for why up to three quarters of new medical devices fail [9]. This issue can be resolved by properly developing requirements, driven by the designated context of use. In the past, several context-driven approaches, such as human-centered design, have been suggested. However, these approaches mostly consist of a few starting points (eg, human-centered design propagates user-involvement from as early as possible). And when they do come accompanied by step-by-step instructions such as SCRUM they are not geared towards the specifics of the eHealth domain. This domain is fundamentally different from other domains such as eCommerce. Therefore, a focus on its specifics is important. In the eHealth domain, the target group for a technology is in most cases known before development starts (eg, patients with Rheumatoid Arthritis, or nurses on an oncology ward). In commerce, a distinctive user group often forms naturally after the introduction of a technology. eHealth developers can and should profile their designated users in detail and should gear design towards this profile, as the end user population can be quite heterogeneous [10]. Next, the relationship between end user and technology provider is a special one. Where a for-profit organization sells a technology directly to a consumer with a limited set of after-sales facilities, an eHealth technology is often offered to insured patients or health professionals as part of a greater service; namely treatment or prevention of a disease or condition. The organization offering this service is then a medical one (eg, a hospital) that bought the technology from the manufacturer. These services are often offered free of charge. This complicates business models that need to satisfy the interests of medical organizations, insurers and external profit, and non-for-profit parties [11]. And as these technologies are part of the treatment or prevention plan, requirements entail more than a list of functionalities only, but also specify how the technology should be embedded in the care context and what the content should convey [12]. Finally, the requirements development approach needs to take into account the boundaries eHealth settings have regarding research options. Care providers may lack time and motivation to participate as their first priority lies with patient care and continuity of care, and workload is generally high. This calls for a well-planned and structured requirements development approach because it is often difficult or impossible to apply endless iterations. In order to deal with these challenges, a dedicated requirements development approach that involves multidisciplinarity is a great asset [7,13].

The current lack of a requirements development approach for eHealth poses several problems. First and foremost, a mismatch between the eHealth technology and the context of use is likely to occur, which can lead to faulty use of the technology, dissatisfaction, low adoption rates, and/or loss of money. Second, it is hard to judge the quality of design activities. It remains unclear which procedures have been followed to collect data to profile the intended end user and to map the designated context of use, and how this data has been translated into eHealth intervention design consequently. Finally, requirements are seldom documented in such a way that they can serve as the basis for evaluations: they are not accompanied by measures for success. This can make it difficult to assess what features or aspects of an eHealth intervention make it effective or not.

In order to deal with domain-specific issues, dedicated requirements development approaches have been introduced in other domains, such as the eGovernment context. Here, the provider of the technology and the user (a citizen, or organization) often hold a contradictory view of the task to be completed and the substeps involved; governments need to design for the mainstream as well as for exceptional situations; users apply, sometimes illegal, workarounds that are necessary for completing a procedure, but which a government cannot design for; etcetera [14]. As a result, several publications have discussed how to deal with these issues in requirements development [15-17]. The eHealth domain has not yet reached this level of maturity.

 This article presents an approach for requirements development for eHealth which incorporates activities from disciplines such as engineering, human-centered design and business, with the goal of creating a human-centered design as well as a business model and implementation plan. Rather than providing an overview of all the possible instruments that one *can* apply here, we provide a hands-on guideline on how to conduct one set of attuned activities. In the next section, we will introduce, the Center for eHealth Research (CeHRes) roadmap, the holistic design approach in which we have embedded our requirements development approach. Then, we discuss its constituent phases. We end this article with a discussion of the (dis)advantages of the approach.

## Methods

### Center for eHealth Research Roadmap

**Figure 1:**
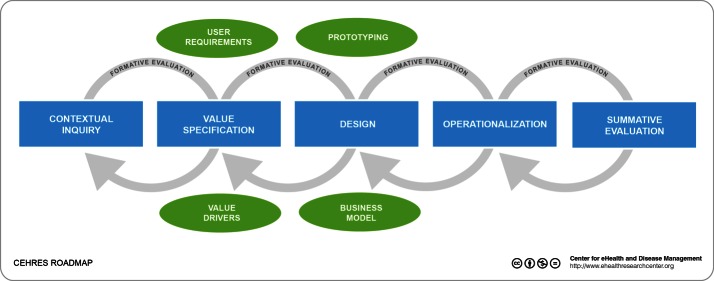
CeHRes roadmap.

The CeHRes roadmap [13,18] is a development approach for eHealth interventions. In order to create value-adding and sustainable eHealth technologies, it incorporates both a human-centered design and a business modeling focus. Human-centered design implies that, prospective, users are consulted throughout the design process, their use of prototypical versions of the system is researched empirically, and iterative design (going through several cycles of design and evaluations) is used [19]. Business modeling focuses on creating an optimum fit between technology, organizational procedures, and organizational resources [11]. Furthermore, the CeHRes roadmap places a strong emphasis on creating persuasive technologies, for example to motivate citizens to conduct healthy behavior. The inclusion of persuasive features in eHealth has been shown to have positive trade-offs such as increased adherence [20]. The roadmap consists of five phases (see Figure 1):

1. *Contextual inquiry*. Here, information on the context of use, the designated end users and the professionals that need to implement the eHealth intervention is collected.
2. *Value specification*. Data from the contextual inquiry is translated into stakeholder values and requirements for the technology.
3. *Design*. Prototypes of the eHealth technology are created on the basis of requirements and tested.
4. *Operationalisation*. The final version of the eHealth intervention is launched and additional resources (eg, user support) are mobilized.
5. *Summative evaluation*. Finally, the uptake and effect of the eHealth technology is evaluated.

Throughout the development process, formative evaluations should be conducted in order to test design assumptions and prototypes. If necessary, developers should revisit a phase in the design process in order to update their insights. This also applies to the requirements development process.

Many factors that determine whether or not an eHealth technology is useful or usable go beyond the interface and interaction design [21], and can only be uncovered when activities aimed at eliciting requirements specifically address the designated context of use [7,22]. Therefore, we present an approach that is founded in the CeHRes roadmap and puts emphasis on the modeling of this context. It is beyond the scope of this article to discuss every possible method for developing requirements. Instead, we will present one possible approach that caters for the demands the health care setting places on creating technology, as we discussed before. It provides the reader with a selection of appropriate and attuned methods out of the huge toolkit and in the end will result in a set of requirements that can lead to value-adding and viable eHealth technology.

The five phases in the requirements development approach within the CeHRes roadmap, their main activities, and the products that are the result of each phase are displayed in Table 1.

**Table 1:** Phases and main activities in the requirements development approach.

CeHRes roadmap phase	Requirements development phase	Main activities	Products
Contextual inquiry	Preparation	Composing the project team	
		Deciding upon the overall goal(s)	
Contextual inquiry	end user and stakeholder identification	Audience segmentation	List of primary end users and stakeholders
		Stakeholder Elicitation	
Contextual inquiry	Requirements elicitation	Conducting interviews, focus groups, observations	Transcripts
Value specification	Requirements analysis	Determining values, attributes and requirements	Values, attributes and requirements
			Personas
Design	Communicating requirements	Completing requirement notation templates	Design document
		Creating the design document	

### Preparation

First, the project team needs to be assembled. As we discussed before, a multidisciplinary design team is a must for coping with the specific demands the eHealth context places on design. The team needs to consist of at least 2 experts in the field of eHealth design and business modeling, 1 relevant medical expert, and, preferably, 1 representative from the programmers. They are responsible for project management and together they have to decide on the overall goal(s) of the eHealth technology. This is also the moment in time where constraints have to be identified (like legal or accessibility guidelines which need to be followed) and an eventual technology push (eg, opting for a mobile eHealth technology as mobile apps will dominate the market soon) has to be decided upon.

### End User and Stakeholder Identification

In eHealth, the end user population can often easily be determined at the start of the development process. However, which end user group is then most important within this population remains unknown. The design team should identify these groups. This way, they know whose characteristics and wishes they should uncover and take into account. Plus, the wide range of stakeholders must be uncovered so that their needs can be accounted for in order to let the implementation of the technology proceed smoothly and to create a sustainable business model.

End users are people who will use the technology directly, like citizens using a mobile app to lose weight or nurses using a teledermatology system [23]. Stakeholders are all the persons or organizations that have a task or role in relation with, or are affected by, the eHealth intervention [24], like organizational purchasers, marketing staff, or a user support department. A person can be both an end user as well as a stakeholder.

#### Audience Segmentation

Profiling the end user in a *professional setting* is often a relatively simple task. The idea for such an eHealth technology is developed with a clear-cut, relatively homogeneous end user population in mind, such as nurses on an oncology ward. In the case of *public eHealth technology*, the profiling of the end user is more difficult. These technologies are designed for patients groups (eg, people with a sleep disorder), or sometimes even for the whole population of a country (eg, a website on when to visit your family doctor) and these are heterogeneous populations. Their motivations for (not) using these technologies or complying with the advice they provide are diverse, and they are people with different cultural backgrounds, skills, and disabilities [15]. In order to deal with the heterogeneity of the end user population of a public eHealth technology, one should identify, profile and design for distinctive audience segments.

Audience segmentation is concerned with identifying homogeneous sub-populations (segments) within a population, and their profiling. In this phase, identifying audience segments is the main goal, profiling is done later on. In order to identify audience segments, one must first uncover the determinants of knowledge, attitudes, and behavior for a given context; preferably from existing research. Then, one must identify audience segments based on distinctive patterns of these determinants [25]. Ideally, the identification of audience segments is based upon the analysis of large sets of quantitative data [26] and uses a combination of demographical, health, and psychographical variables [27]. For a more thorough discussion of audience segmentation we refer to Slater [25].

#### Stakeholder Identification

Stakeholder identification aims at creating a list of stakeholders that need to be involved in the design of the eHealth intervention. In the literature, several lists of variables such as [24,28] and frameworks, as cited in [29,30], can be found that serve as input for thinking about who to include as stakeholder. However, a clear-cut and relatively simple procedure is missing. The approach we suggest to identify stakeholders consists of four steps:

1. A first inventory of relevant stakeholders is created based on the relevant protocol(s) or clinical pathway(s) for a given context. One should scrutinize the documents to identify actions and the person(s) or organization(s), responsible for each action. If these documents are not available, one can hold a brainstorm session with the client.
2. This inventory is then checked with the client and/or an expert in the field. Are the identified stakeholders correct? Which stakeholders are missing?
3. If the list of identified stakeholders is too long, a selection is made, based on an estimation of each stakeholder’s power, legitimacy, and urgency. A combination of these factors make up their salience [24].
4. These stakeholders are invited for a stakeholder session (see section on requirements elicitation). During this session, the role(s) each stakeholder plays in the prevention or treatment of a condition or disease is mapped (see [31] for questions that can guide this discussion). On the basis of this map, the stakeholders discuss which stakeholders are missing, thereby creating the final overview. If new, important stakeholders are identified, they need to be interviewed about their role in the prevention or treatment.

As protocols or clinical pathways are not always very clear on the role each stakeholder plays in a given context, it is important to validate the list that results of step 2 and 3 with the stakeholders themselves, as we suggest in step 4.

### Requirements Elicitation

Now that one has identified the end users or end user segments for an eHealth technology, it is time to profile them and to map their context of use. The identified stakeholders need to be consulted in order to map the current prevention or care path for a given condition or disease, and the opportunities and barriers for the eHealth technology and its implementation. By focusing on these matters one can determine what the eHealth technology needs to do and how it should be implemented. Requirements elicitation methods provide the tools to elicit the necessary input.

One kind of knowledge that is important to uncover during the requirements elicitation phase, is so-called ‘tacit knowledge’. This kind of knowledge is “neither expressed nor declared openly but rather implied or simply understood and is often associated with intuition” [32]. Mostly, this consists of steps taken in routine tasks; like comforting a distressed patient. These tasks do not consist of predefined steps which are easy to explain to somebody. Rather, it is something one ‘just does’. This makes it a difficult procedure to map. However, it is an important activity, as it is crucial that the features and interface and interaction design of an eHealth technology are in line with the end users’ tacit knowledge. Several methods can be applied to elicit tacit knowledge; like observing potential end users, or asking them to tell stories about typical tasks or occurrences on the job (for a complete overview see [33]). Regardless of the method one uses, it is important to determine before data collection how to go about eliciting tacit knowledge since it will not be handed to the project team on a plate.

There is a wide variety of requirements elicitation methods, each with their own strengths, and limitations (for overviews, see [34,35]). We will shortly address the three most popular methods:

- *Interviews* may be used to uncover end users’ or stakeholders’ behavior or opinions, their motivations or rationale for these, and their wishes regarding the to-be-developed eHealth technology. They are also well-suited for collecting data upon which personas can be based (see [36]). Personas are fictitious users whose characteristics resemble the average for an end user (segment) and who is presented in a biography with a photo [37]. Personas are well suited in this context, as they are easy to understand for the wide variety of stakeholders involved in eHealth design. They can then be used to spark the discussion among stakeholders during a focus group or can serve as input for content requirements. Interviews should be used to profile end users and stakeholders, and to elicit requirements that will specify functions, content, and the user experience. For more information on how to conduct a requirements elicitation interview, refer to [38].
- *Focus groups* can be used for establishing the context, roles and primary tasks that are or could be supported by technology with stakeholders, and what business model should support this. Via personas, scenarios and task demonstrations, stakeholders can gain insight into, and reach consensus on the context, the division of roles, the scope of the eHealth technology, the flow of funds, requirements, and requirement priority. Focus groups can also serve to explore the context and need of a new activity or work practice that involves eHealth, to learn how this could be designed and introduced into current work patterns or daily activities [39]. Again, personas and scenarios may be used to elicit ideas on the new activity. In short, focus groups are very well suited to elicit input for implementation strategies and business models. For instructions on how to conduct focus groups, refer to [40].
- *Observations* can be especially useful for understanding actual end user behavior and their social, physical and spatial surroundings [41]. As a result, they also provide the option to see what tacit knowledge drives end users. Observations can be used to elicit requirements that specify the functions and modality of the eHealth technology. For more information on observations, see [42].

### Requirements Analysis

Once requirements elicitation sessions are completed, their output needs to be translated into requirements. This step often remains unmentioned in requirements engineering reports, and methodological explanations of this step are scarce. We hereby present a method for translating raw data into requirements, based on [43]. In this method, for each part of a transcript that is worthy of translation into a requirement, three derivatives are determined: values, attributes and requirements.

- *Value* is an ideal or interest a (future) end user or stakeholder aspires to or has.
- *Attribute* is a summary of the need or wish that is spoken out by the (future) end user or stakeholder.
- *Requirement* is a technical translation of an attribute.

Each derivative can be used to communicate about the eHealth technology with a specific group of people (technologists understand requirements, marketing departments use attributes, and policy makers prefer values). Furthermore, attributes and values can be used to group requirements, which makes it easier to set priorities later on.

The basis for the translation process are the transcripts created from the requirements elicitation sessions (eg, the typed-out interviews). One issue that first needs to be resolved is to determine what counts as something that should be translated into a requirement. It is impossible to formulate fixed rules for solving this dilemma. And tempting as it may be, focusing on prevalence does not guarantee success. When an issue is often brought forth, it does not mean it needs to be translated into a requirement (eg, it may be an issue that should be resolved by creating new legislation). If an issue is brought forth only once, it is possible that it provides a great contribution to the eHealth technology. Rather, we follow Braun and Clarke [44] and suggest that an issue should be translated into a requirement when it captures something important in relation to the overall goal(s) of the eHealth technology.

To aid the translation process, the analyst can complete a translation table. The translation table shown in Table 2 is filled with data from the development project of bedside technology to aid hospital nurses in making prudent and correct use of antibiotics. The following steps should be taken to ensure a reliable translation of data into requirements.

1. The analyst familiarizes him or herself with the data.
2. Quotes that capture something important in relation to the overall goal(s) of the eHealth technology are identified and listed in the “user expression” column.
3. For each quote, the attribute or attributes are determined. An attribute should be formulated as a very short summary of the end user or stakeholder expression.
4. Quotes are grouped on an attribute level. Quotes that can be transformed into the same attribute are merged in one row.
5. The analyst checks all quotes and the attributes that flow from them, and determines whether the attributes are correct and distinctive. If necessary, attributes are adjusted.
6. Per attribute, one or more requirements are formulated. They specify the end user or stakeholder expression into terms a system designer can work with. Requirements should be formulated as precisely as possible, and usually are sentences like ‘The system must…’
7. An independent analyst checks the attributes and requirements formulated. He or she notes disagreements or suggestions. Then, the initial and second analyst discuss these findings.
8. Attributes and requirements are adjusted based on the discussion between the first and second analyst.
9. The first and second analyst determine the values together. Most often, there are only a few values that are linked to many attributes. Values should be formulated in a few words.

Once the translation table has been completed, the requirement templates can be filled out (see Section for Completing requirement notation templates). At the same time, personas can be constructed on the basis of the raw data (for a stepwise procedure, see [45]). These personas can then be used to formulate content requirements, or as input for stakeholder sessions.

**Table 2:** Data analysis table for antibiotic stewardship app.

	User expression	Value	Attribute(s)	Requirement(s)
	Nurse 1: “But wouldn’t it be nice if you have the medications in the electronic prescriptions system, and that you can click on the medication and just click on through.” Pharmacist: “that you can instantly…” Nurse2 : “directly…” Nurse3: “yes, that it is available directly” Pharmacist: “Yes, for prescribing [a medicine] I can imagine that he [the physician] needs the information from an indication-point of view. And for you I can imagine that you would want to have the information focused on the application; how to do it all.” Nurse 3: “What should I pay attention to.”	Easy access	One-stop-portal for information	The system incorporates data from databases for patient and protocol/procedural information The system provides access to all (types of) information via one interface

### Communicating Requirements

#### Completing Requirement Notation Templates

**Figure 2:**
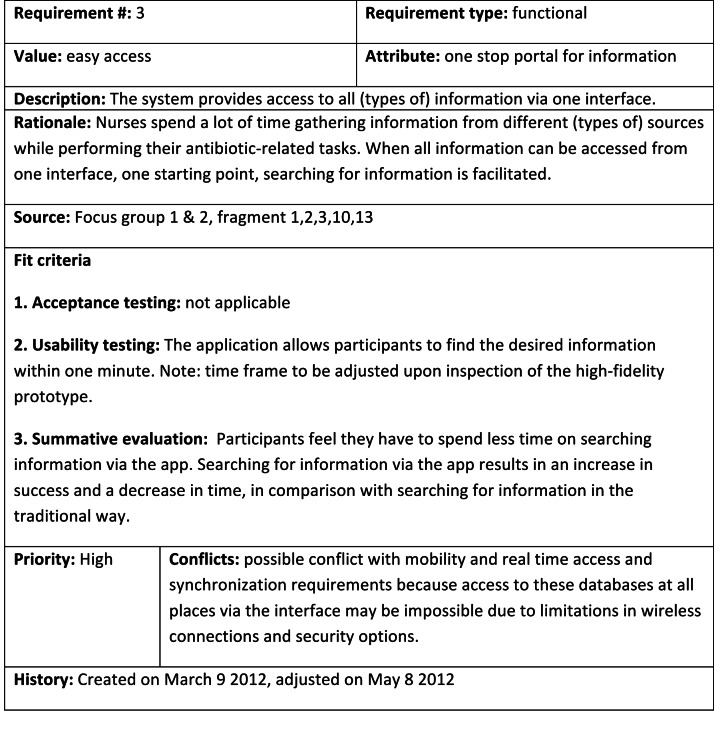
Completed requirements notation template.

At this point, the project team will have a list of requirements, derived from elicitation activities. These should be expanded with requirements, derived from relevant literature (like persuasive design tactics when persuasive technology is developed), legal constraints, and demands on accessibility. Each requirement needs to be documented in such a way that it enables programmers to understand what needs to be made and why. Requirements documentation should also serve as the starting point for evaluations (both aimed at generating redesign input, and aimed at assessing the effect or return-on-investment). We created a requirements documentation template, based upon the Volere template [46], as it supports the aforementioned goals. The template is depicted in Figure 2 and completed for one requirement from the same development project on bedside technology for hospital nurses.

##### Requirement Number

Each requirement is assigned a unique ID.

##### Requirement Type

There are different kinds of requirements that need to be shared with different kinds of people that are involved in the creation of the eHealth technology. We discern these types:

- *Functional and modality requirements* specifying technical features and on what kind of technology (eg, tablet, smartphone or desktop PC) and operating systems the technology should work. Mostly meant for programmers.
- *Service requirements* specifying how services surrounding the technology, like marketing or user support, need to be organized. Mostly meant for managers, responsible for these services.
- *Organizational requirements* specifying how the technology should be integrated in the organizational structure and working routines. Mostly meant for managers of the organizations in which the technology is to be used.
- *Content requirements* specifying the content that needs to be communicated via the technology and, if applicable, language level, persuasive approach, and special accessibility demands. Mostly meant for content managers.
- *Usability & User experience requirements* specifying the interface and interaction design of the technology and how user experience factors, such as trust or fun, should be integrated into the technology. Mostly meant for human factors specialists.

##### Value, Attribute, and Description

Here, the value, attribute, and description (the requirement itself) are noted down.

##### Rationale

Each requirement is accompanied by a short statement justifying the need for this requirement, preferably linked to a source. The rationale must convince a programmer that the requirement is worthy of inclusion.

##### Source

The source(s) of each requirement (eg, the interview number or persona) is noted down for reference purposes.

##### Fit Criteria

Requirements are a translation of end users’ and stakeholders’ needs and wishes into design, and should therefore be checked. Fit criteria are measures of success for this translation and are the basis of evaluations. Often, functional requirements cannot be evaluated with users as they are simply implemented or not (like a requirement specifying that type of data x is collected from database y). In this case, formulating a fit criterion is useless. In the other cases, whether or not a fit criterion is formulated or not depends on its priority (when there is no possibility to evaluate all requirements, only those with a high priority, or controversial requirements should be evaluated), and whether or not the prototypical version of the system that will be used supports evaluating the fit criterion (eg, testing for usability with a simple prototype will yield very limited results). Roughly, we discern 3 kinds of evaluations.

###### Acceptance Testing

By demonstrating a very simple prototype (eg, paper and pencil sketches) that demonstrate the main functionality and look & feel of a technology, and its associated working routine, user and stakeholder acceptance of crucial or controversial features can be determined early on [47]. Based on this evaluation, the inclusion of these features should be settled. A fit criterion should tell when the feature or working routine, specified in the requirement, is considered to be accepted.

###### Usability Testing

By making end users or experts interact with a clickable prototype that approximates the final version of the technology in terms of functionality and interface & interaction design, usability issues can be found [48]. Typical usability evaluation methods, such as heuristic evaluation, cognitive walkthroughs or thinking-aloud, can support the elicitation of these issues [49]. This evaluation drives the modification of the interface & interaction design of the technology. A fit criterion should tell when a requirement is translated in a usable manner.

###### Testing for Effect

With the version of the eHealth technology that is launched, its effect and return-on-investment can be assessed. Often, it is difficult or impossible to determine the effect of an eHealth technology on its overall goal. For example, proving that a reduce in general practitioner consultations for tick bites is due to a mobile app that instructs people how to prevent or deal with tick bites, is impossible to do. Many factors outside the mobile app will play a role and it is extremely difficult to map all of these factors, and to establish causal links to the number of general practitioner visits. Therefore, following the concept of attribution theory [50], evaluations of eHealth technology should focus on outcomes on a lower level, that can be linked directly to a specific feature, and indirectly to the overall goal(s) of the eHealth technology. The fit criterion field in the requirements template forces the project team to use the requirement for the formulation of feature-specific effect measures. Several methods, like data log analysis and user surveys (for a full overview, see [51]) can be useful here.

We do not think that all requirements should be evaluated, or should be evaluated at all 3 instances. We advocate the evaluation of requirements with a high priority, or controversial requirements (like those to do with privacy). When a fit criterion is not met, the (prototypical) system should be redesigned and re-evaluated. 

##### Priority

Often, not all requirements that are elicited and formulated can be realized in the design. Limited resources, like time and money, force the project team to make a selection. In the literature, many approaches are described that guide the prioritization process (for an overview, see [52]). However, these methods often demand from the project team that they consult their stakeholders and end users repeatedly, and often use complicated metrics. For the design of large-scale eHealth systems (like a national electronic patient file) one should apply these methods in order to deal with the large number of different stakeholders and limited budgets. However, for the scope of many eHealth projects, these approaches are too time-consuming and complex. Therefore, we recommend to set stakeholder and requirement priority by a discussion among the project team members. They should distinguish stakeholders and requirements with a high, medium and low priority. When ranking stakeholders, their power, legitimacy, urgency and salience should be taken into account [24]. When determining the priority of a requirement, the following should be considered: the priority of the associated stakeholders, its importance, the penalty for not implementing the requirement, cost, lead time, risk, and volatility [52].

##### Conflicts

If applicable, conflicts with other requirements should be listed here. The project team should find a solution to a conflict, and must translate this into a new requirement, or one requirement should take precedence over the other, based on priority.

##### History

In this section, it should be documented how the requirement is translated into design, or the reasons why it was omitted. Furthermore, changes to the design because of evaluations should be listed, as well as scores of effectiveness measures. This way, a complete overview of a requirement’s origin, translation into design, and effect can be created.

## Results

### Creating the Design Document

Once all requirements are documented in templates, the design document can be created. It is important that such a document is made for several reasons, like making it possible to estimate the costs of creating the technology, preventing programmers from making their own requirements, and preventing a brain drain if a project team member leaves the project [53]. This document must allow the people that need to program or implement the eHealth technology to do so. Therefore, it has to include an overview of the eHealth technology goals, requirements and a low-fidelity, or paper, prototype. It must also include sections that specify the technological design of the technology, such as entity-relationship diagrams or dataflow diagrams. Besides creating a design document, we recommend to also present the directives in person to programmers and involved managers. Finally, the information gathered from the different stakeholders, must serve as input for an implementation plan and business model.

## Discussion

### Principal Findings

In this article we have presented a multidisciplinary requirements development approach for eHealth design. Its main aim is to support the creation of context-driven eHealth technology that matters by applying a human-centered, context-driven design approach that includes the creation of an implementation plan and business model. The approach supports the identification and profiling of end user groups and stakeholders, forces the project team to identify requirements in an empirical manner, and advocates the formulation of feature-specific effect measures for the eHealth technology. The latter allows researchers and policy makers to learn exactly why an eHealth technology does or does not live up to its expectations.

In the literature and practice, several other approaches to requirements development are often discussed and applied: agile design (eg, SCRUM), participatory design, and more technical approaches (eg, RUP). Agile design shows quite some overlap with our approach, as it makes use of iterative design cycles in which the prospective end user is a focal point for design. The downside of agile design approaches is that they do not take the organization into account and hence, do not support the development of an implementation plan and business model [54]. Our approach does provide a basis for the generation of these. In participatory design, the end user and other stakeholders play an active role in the design team [55]. This way, their view and context are brought into the design. This approach is somewhat similar to human-centered design and we think it is certainly possible to incorporate activities from participatory design into our approach. For example, a design workshop in which end users and the development team create a first prototype together would be a very suitable method for the requirements elicitation phase to generate design ideas, and to elicit the aspects of the end users’ context that need to be taken into account. However, the literature on participatory design often fails to provide hands-on guidelines on how to apply a method. Our approach guides the project team in detail. Finally, the technical approaches, such as RUP, are very limited in their capabilities to incorporate the needs, wishes and organizational context of the end user into design [56]. Our approach is the first to take into account the specifics of eHealth technology and to overcome the limitations of the popular requirements development approaches. Furthermore, this approach allows a great degree of freedom for choosing the most suitable method for activities like identifying end user sub-groups and requirements elicitation. We feel this is important as each development process is unique (in terms of time, budget or the amount of research on which the development builds forth) and the methods one uses should be geared towards the development context.

### Limitations

The approach to requirements engineering we have presented has some downsides. First, because it is very thorough it takes quite some time and effort to go through all the steps. This critique has been voiced towards many requirements engineering approaches, and several faster and less thorough, or agile, approaches have been proposed as a counter reaction. Agile approaches advocate the development of technology with a small team of experts and customers, and the rapid development of prototypical versions of the eHealth technology which are evaluated and redesigned [57]. However, as we discussed above, agile development does not take into account the implementation plan and business model. Furthermore, agile design may not always be possible in health care settings, where research activities can demand too much time, or can put too much emotional constraints on health care workers or patients, whose first and greatest priority lies with getting well or providing good care and not in participating in eHealth development activities. Consulting them repeatedly about the technology in a short time-span may prove to be impossible. Being well-prepared and having a thorough plan like the approach we describe here, adds to development efficiency: it allows designers to get the maximum out of each stakeholder or end user consultation. Maybe for this reason, the application of agile approaches is not widely adopted yet. We encourage project teams that do opt for an agile design approach, to still utilize a structured manner of data analysis and requirements notation, as we have set out in this article. A second downside of our approach is that it requires the use of specialists in requirements elicitation and notation. Conducting a useful requirements elicitation interview, or constructing good requirements with proper fit criteria is not an easy task and requires a lot of experience. Therefore, we advocate the inclusion of an experienced requirements developer in the project team.

The danger of consulting end users and stakeholders, and making their voices and interests the primary focus of the to-be-developed eHealth intervention, is that it limits creativity [58]. It is therefore important to find a balance between end user and stakeholder input, and creative ideas from the design team. The latter should not necessarily be made subordinate to end user and stakeholder input, but should be given a fair chance in acceptance and usability tests. Participatory design sessions with end users or other stakeholders can release this creativity [59]. Resulting creative solutions can then be noted down as a requirement or several requirements in the requirements notation template. However, creativity in eHealth design is a topic that has not been paid enough attention to in the design literature to date. Future research should delve into it and determine its place and value in eHealth design processes. Furthermore, methods for identifying and involving organizational stakeholders into the design of eHealth are often very comprehensive and time-consuming. The development of lightweight methods for these goals would be a welcome addition to the requirements developer’s toolkit.

### Conclusions

We hope that this article will inspire eHealth technology designers to apply a more systematic approach for their requirements engineering activities. This is most likely to have beneficial consequences for the eHealth technology (in terms of costs, usefulness, adoption, etc), as well as for the community as a whole. We also encourage researchers to report case studies of their requirements development experiences (either guided or not guided by our approach). This way, we will be able to estimate the worth of different requirements development approaches for the eHealth domain and the benefits and downsides of the design methods used.

## Abbreviations

CeHRes: Center for eHealth Research
